# Speech Development Between 30 and 119 Months in Typical Children III: Interaction Between Speaking Rate and Intelligibility

**DOI:** 10.1044/2024_JSLHR-24-00356

**Published:** 2024-12-16

**Authors:** Tristan J. Mahr, Paul J. Rathouz, Katherine C. Hustad

**Affiliations:** a Waisman Center, University of Wisconsin–Madison; b Department of Communication Sciences and Disorders, University of Wisconsin–Madison; c Department of Population Health, Dell Medical School, University of Texas at Austin

## Abstract

**Purpose:**

Earlier work has established developmental benchmarks for intelligibility and articulation rate, but the intersection of these two variables, especially within individual children, has received limited attention. This study examines the interaction between intelligibility and speaking rate in typically developing children between the ages 2;6 and 9;11 (years;months) and evaluates whether children show a speed–accuracy trade-off in their habitual speech production.

**Method:**

Speech samples of varying lengths were collected from 538 typically developing children. Intelligibility was measured as the number of words correctly transcribed by untrained adult listeners, and speaking rate was calculated in number of syllables per second. Regression models estimated the effects of age, utterance length, and speaking rate on intelligibility.

**Results:**

Intelligibility and speaking rate were positively correlated overall but weakly correlated after adjusting for age. In regression analyses, intelligibility increased with age and decreased with utterance length, and there was a trend for intelligibility to decrease with increased speaking rate, especially in longer utterances. At the individual level, for most children, there was a negative effect of speaking rate on intelligibility.

**Conclusions:**

Our findings provide evidence from a large-scale sample for the hypothesis that children's speech is subject to a speed–accuracy trade-off where increased speaking rate leads to reduced articulatory accuracy and hence reduced intelligibility. Further research is needed on how to apply this trade-off in a clinical setting.

**Supplemental Material:**

https://doi.org/10.23641/asha.27964125

Speech development occurs over a protracted period of time, beginning in infancy and ending at some point in adolescence for neurotypical children. Many factors drive the development of speech including anatomical growth of the vocal tract (Vorperian et al., 2005, 2009; Werner et al., 2021), physiological changes across subsystems (Boliek et al., 1997; Hoit et al., 1990), refinements in speech and motor control (Connaghan et al., 2004; Green et al., 2000), and synergistic cognitive and language developments (Nip & Green, 2013; Nip et al., 2009, 2011). The development of speech sound articulation in particular has received considerable attention, both in historical literature (Conradi, 1904; Poole, 1934) and in recent literature (Crowe & McLeod, 2020; McLeod & Crowe, 2018).

The study of speech intelligibility has received increasing large-scale attention from a developmental perspective. In developing the Test of Children's Speech (TOCS+; Hodge & Daniels, 2007) and establishing its validity, Hodge and Gotzke (2014) examined intelligibility and speaking rate in typically developing English-speaking children (*n* = 48, ages 3–6 years). For this sample, average intelligibility scores increased with age, and variability among children (*SD*s) in each age group tended to decrease with age. Average speaking rate also increased with age, but there was not a clear age-related decrease in variability.

We extended the work of Hodge and Gotzke (2014) by examining the development of speech intelligibility (Hustad et al., 2020, 2021) and articulation rate (Mahr et al., 2021) in a cohort of over 500 typically developing English-speaking children between the ages of 30 and 119 months. In these studies, we established normative standards for intelligibility acquisition and articulation rate development in the form of percentile growth curves, laying a foundation for the identification of children who do not meet age-level expectations (Hustad et al., 2023) and who may therefore require further assessment and subsequent intervention. Key findings from these studies show that both intelligibility and articulation rate increase with age. Both are still developing well into middle childhood for children who are below the 50th percentile of age expectations. Furthermore, intelligibility and articulation rate show marked within-age between-child variability and faster growth at early ages followed by slower growth later in development. Notably, between-child variability markedly decreased with age for intelligibility. Utterance length also had an effect such that articulation rate increased with utterance length; after the age of 40 months, intelligibility was higher for multiword utterances than for single-word utterances.

Our findings are consistent with studies examining intelligibility and rate within a cohort of German-speaking children. Specifically, Schölderle et al. (2021) performed a similar set of analyses to examine the development of intelligibility and articulation rate in typically developing German-speaking children (*n* = 144, ages 3;0–9;11 [years;months]). Intelligibility growth was steep at young ages, and variability among children followed an inverted funnel-like pattern, marked by greater variability at younger ages that decreased as children got older. Articulation rate increased with age more gradually, and between-child variability did not noticeably decrease with age. These authors also examined communicative efficiency, by taking the product of rate and intelligibility to yield intelligible syllables per minute. Not surprisingly, results showed that development of communicative efficiency followed a trajectory that combined features of articulation rate and intelligibility (i.e., steeper growth than articulation rate and more variability than intelligibility).

Although this communicative efficiency gives us a glimpse into how rate and intelligibility intersect, Schölderle et al. (2021) did not examine the extent to which rate and intelligibility interact within age levels. Thus, we do not know whether children of the same age who speak more quickly are more intelligible or less intelligible than children who speak more slowly. This intelligibility–rate interaction may have important clinical implications given that rate reduction is a clinical strategy for improving intelligibility in some populations such as those with dysarthria (Sakash et al., 2020; Yorkston et al., 1990). Rate reduction may support intelligibility by allowing a listener more time to process speech (Blanchet & Snyder, 2010), and it can have more direct acoustic consequences such as increased vowel area (Hustad & Lee, 2008; Tjaden & Wilding, 2004). Reduced speaking rate also emerges as a byproduct of interventions in children that target clear speech (Levy et al., 2017). By studying the relationship between rate and intelligibility in typical children, we can understand incidental speed–accuracy trade-offs as a baseline of comparison for deliberate, clinical manipulations of speaking rate.

One hypothesis is that increased speaking rate will result in reduced intelligibility because speech is subject to a speed–accuracy trade-off. For example, Lammert et al. (2018) formally apply this idea (Fitts' law) to speech magnetic resonance imaging data. Results showed that movement time between nucleus–coda phoneme pairs correlated with a difficulty metric based on the distance between phonemes and narrowness of the phoneme targets, as predicted by a formal speed–accuracy trade-off. The authors also found that these correlations were themselves negatively correlated with speaking rate for individual speakers: Speakers who showed stronger correlations between movement time and phoneme-pair difficulty had slower speaking rates.

It is also possible that children with faster habitual speaking rates have more developed speech-motor skills overall, and so these children will have higher intelligibility than other children their age. Redford (2014) examined listener ratings of speech clarity in 5- to 8-year-old children, finding that children with faster habitual speaking rates received higher ratings of speech clarity even when controlling for age and utterance length. One interpretation of these results is that clear articulation and a fast speech tempo both draw on speech-motor control, so children with more developed speech-motor skills do both (speak more clearly and speak more quickly). We note, however, that articulatory clarity and speech intelligibility are distinctively different constructs. Intelligibility is measured at a lexical level, while articulatory clarity describes speech at a segmental level.

In the present study, we examine the effect of speaking rate on intelligibility over and above the effects of age and utterance length. This research article is the third in a series (see Hustad et al., 2021; Mahr et al., 2021) focused on quantifying (cross-sectional) developmental change in functional measures of speech in typical children. Figure 1 provides a graphical version of our key analysis variables. We posit that there is a latent speech-motor control variable that influences both speaking rate and intelligibility. However, because we cannot directly measure speech-motor control in the available data set, we use age as a proxy for (the development of) speech motor control. In our prior work (Hustad et al., 2021; Mahr et al., 2021), we found that utterance length predicted articulation rate and that children's length of longest utterance predicted intelligibility, so utterance length is a variable of interest for the present study. Age and utterance length both affect speaking rate and intelligibility, so they are confounders that we control for in our analyses. The remaining path is the connection between speaking rate and intelligibility and represents the effect of speaking rate on intelligibility.

**Figure 1.**
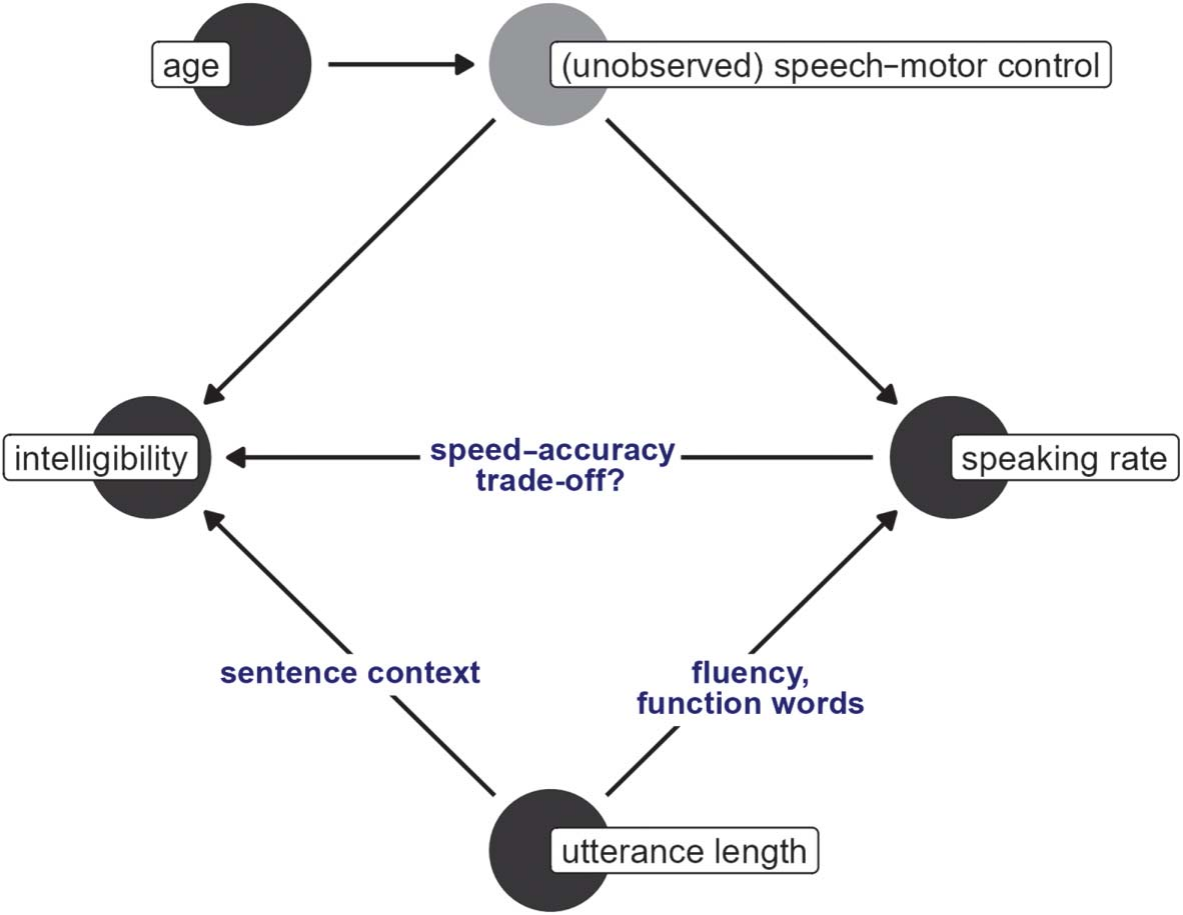
Graphical version of analysis variables. We cannot observe speech-motor control, so we treat age (developmental time) as a proxy for speech-motor control. The path from speaking rate to intelligibility is the effect of speaking rate on intelligibility, adjusting for age and utterance length.

We hypothesize that intelligibility and speaking rate will be highly correlated between children because both measures are known to increase with age and with utterance length. Both intelligibility and speaking rate are speech-motor skills that improve with development (age), so they will be positively correlated. We expect the relationship between speaking rate and intelligibility to change after we adjust for age. Specifically, we hypothesize that increased speaking rate will lead to reduced speech intelligibility in children, particularly for those children who are younger (i.e., less mature in terms of speech-motor control). That is, children who are younger will have less mature speech-motor control and will be more susceptible to a speech-motor trade-off where intelligibility is reduced at faster speaking rates. Older speakers in contrast with more mature speech-motor control will not demonstrate the speed–accuracy trade-off for this task.

In the present study, we addressed the following research questions:

1. How are intelligibility and habitual speaking rate in typically developing children correlated overall and after adjusting for age?
2. What is the effect of speaking rate on intelligibility both between children and within children at different ages?

## Method

As noted, this study is a companion paper to Hustad et al. (2021) and Mahr et al. (2021). The former developed normative growth curves for intelligibility in a large cohort of typically developing children, and the latter developed normative growth curves for articulation rate in the same cohort (plus some additional participants). Because of the overlap with the prior two studies, we provide a condensed methodological description here. This study was reviewed and approved by the University of Wisconsin–Madison Institutional Review Board (Social and Behavioral Sciences [2016-0574, 2018-0855]); informed consent was obtained for all participants.

### Participants

As reported in our companion papers, the community-based sample of typically developing children met the following inclusion criteria: (a) age between 30 and 119 months (2;6–9;11), (b) American English as the primary language in the home, (c) hearing within normal limits per behavioral screening results, (d) speech within normal limits per standardized articulation testing, and (e) language within normal limits per standardized language testing. Children were excluded if they were receiving intervention services for any educational or developmental concern or had any medical diagnoses related to development.

Data from 538 typically developing children were used in the present study. See Hustad et al.'s (2021) study for detailed demographic information on the same children. Briefly, children were 281 biologically assigned females and 257 biologically assigned males. Participants included 107 three-year-olds (i.e., 36–47 months), 112 four-year-olds (48–59 months), 103 five-year-olds (60–71 months), and 101 six-year-olds (72–83 months). There were additionally 56 children under the age of 3 years (30–35 months) and 59 children aged 7 years and older (84–119 months). Most children (87%) were White and from middle/upper-middle–socioeconomic status homes, reflecting the demographics of Madison, Wisconsin, where the study was conducted. The lack of diversity in our sample is a key limitation of this research.

Adult listeners made orthographic transcriptions of speech samples from children, which were used to obtain intelligibility scores. Two different adults with normal hearing heard each child, for a total of 1,076 listeners. Listeners were 280 self-identified men and 796 self-identified women. The mean age of listeners was 20.8 years (*SD* = 3.7). Listeners were paid or received course credit for their participation.

### Materials and Procedure

Materials and procedures for this study are the same as reported in Hustad et al.'s (2021) and Mahr et al.'s (2021) studies. Briefly, all children produced the same corpus of utterances from the TOCS+ (Hodge & Daniels, 2007) using standard elicitation procedures. Recordings of children's speech were made in person in a sound-attenuating suite using a professional-quality digital audio recorder (Marantz PMD570) at a 44.1-kHz sampling rate (16-bit quantization) and a condenser studio microphone (Audio-Technica AT4040) positioned 18 in. from the child's mouth.

For this study, we focus only on utterances between three and seven words in length. Children produced 10 utterances of each length; however, some children were not able to produce all utterances. Productions were included in our analysis corpus, if the children generated at least five utterances of a given length. There were 60 children with a longest utterance length of three words, 56 with four words, 36 with five words, 14 with six words, and 372 with seven words.

### Intelligibility

Intelligibility was measured by having two different naive adult listeners transcribe each child's speech in a controlled in-lab listening experiment (*n* = 1,076 listeners; two per child). Listeners were presented speech samples in a sound field and were asked to type what they thought the child had said for each utterance. Each listener only heard samples from only one child during a listening experiment. Intelligibility was the proportion of words correctly transcribed by a listener on a speech sample, averaged across the two listeners per child. We aggregated intelligibility by child and utterance length so that each child had an average intelligibility score for each utterance length.

### Speaking Rate

Although Mahr et al. (2021) examined articulation rate, the present study considers speaking rate instead. Articulation rate excludes pause time from an utterance's duration, and speaking rate includes these pauses. We favored speaking rate because it is a simple and transparent measure of speech tempo that can easily be computed clinically without need for defining, identifying, or measuring pauses. Furthermore, speech rate is ecologically valid, reflecting the full duration of child talking time that the listener experienced. Speech rate was also highly correlated with articulation rate, with correlations ranging from .958 for seven-word utterances to .996 for three-word utterances.

We computed speaking rate as the number of syllables per second of speaking time. Speech samples and their transcripts were force-aligned with the Montreal Forced Aligner (McAuliffe et al., 2017) to create Praat textgrids, which segmented the start and end of words and phones in each sample. Speaking time was the duration of time from the onset of the first word to the offset of the final word. (Put differently, the duration of the recording minus any initial or final silences.) Number of syllables was computed by counting the number of vowels in the alignment textgrids. (The pronunciation dictionary used for forced alignment did not use syllabic consonants, so this approach does not undercount syllables.) We aggregated these speaking rate measurements by child and utterance length so that each child had an average speaking rate measure for each utterance length.

## Analysis

As noted in the introduction, our analysis was structured around the variables and relationships in Figure 1, where both intelligibility and speaking rate are variables governed by an unobserved factor of speech-motor ability and both can be influenced by utterance length. As a starting point for our regression analyses, we modeled how intelligibility changed with age (as a proxy variable for speech-motor development). We used a beta regression family because intelligibility scores are proportions. The beta regression included a mean component (i.e., estimates the average intelligibility on the log-odds scale) and a dispersion component for the precision parameter. Age was included in the mean component using a 3-*df* natural cubic spline and in the dispersion component using a 2-*df* natural cubic spline. Thus, the expected intelligibility and the precision could both change flexibly with age. The data set featured repeated measurements (one intelligibility score per utterance length per child), so we included by-child random intercepts to account for the within-child correlation of intelligibility scores.

We included main effects of utterance length and speaking rate. These parameters estimated how the intelligibility of a typical child would change with utterance length and change with speaking rate. Because of the design of the task, where sentences are administered in batches of 10 items per utterance length, utterance length here was a methodological artifact, reflecting both the number of words in the utterance and general progression along the speaking task. So, we treated utterance length as a dummy-coded categorical variable and included Speaking Rate × Utterance Length interactions. That is, there was a baseline set of model parameters for three-word utterances, and parameters for four-, five-, six-, and seven-word utterances were adjustments made to this baseline. We assessed an alternative model where utterance length was treated as a monotonic variable (Bürkner & Charpentier, 2020). That is, there was a general length effect that stayed the same or increased in magnitude at each step in utterance length. Model comparison favored the model with categorical length. Specifically, the monotonic model had a much smaller number of effective parameters (monotonic vs. categorical: 523 vs. 852), but the categorical model had better expected predictive accuracy (monotonic expected log predictive density [ELPD] = 3,715 vs. categorical ELPD = 3,814).

Our baseline model, therefore, included a flexible age effect, a linear speaking rate effect, categorical effects of length, and interactions between speaking rate and utterance lengths, and it included by-child random intercepts. We augmented this model to examine other random-effects configurations in a stepwise manner. First, we fit two alternative models that (a) included by-child random effects for utterance length or (b) included by-child random slopes for speaking rate. We then tried three additional models that included random slopes for both rate and utterance length in different configurations (c, d, e). We compared the models with approximate leave-one-out cross-validation with the loo R package (Version 2.7.0; Vehtari et al., 2017). Model comparison favored Model (a) over any model that included by-child speaking rate effects, so we report that model in our analysis results.

We fit the model using a Bayesian approach. We used weakly informative priors and Hamiltonian Monte Carlo (HMC) on four chains to obtain a total of 6,000 post-warmup posterior draws. We chose this Bayesian approach because it allowed uncertainty to propagate through all estimates and comparisons. Each posterior draw provided a plausible set of parameter estimates, so for any estimate—for example, what is the effect of rate on three-word utterances for 4-year-olds—we could compute the estimate on each posterior draw to obtain a distribution of plausible values for that estimate.

Because the coefficients from the model are difficult to interpret (log-odds values for a child with a random intercept of 0), we report results using the population-average (marginal) means for intelligibility on its natural (proportion) scale. That is, on each posterior sample, we computed subject-specific fixed-effects means (i.e., predictions for a typical child on the log-odds scale) and then averaged over between-child variability by sampling/simulating 1,000 new children using the covariance matrix of the model's random effects. Averaging over these 1,000 simulated children on the proportion scale provides an estimate for the population mean.

Analyses were carried out in the R programming language (Version 4.4.0; R Core Team, 2024). HMC sampling was performed using Stan (Version 2.34.1; Stan Development Team, 2024) through the brms R package (Version 2.21.0; Bürkner, 2017) and the cmdstanr backend (Version 0.7.1; Gabry et al., 2024). Supplemental Material S1 provides analysis code.

## Results

### Correlation of Intelligibility and Speaking Rate

Simple Pearson correlations between speaking rate and intelligibility ranged from .18, *p* < .001, for five-word utterances, to .28, *p* < .001, for four-word utterances. These positive correlations are confounded by age, which affects both intelligibility and speaking rate. Therefore, we computed age-residualized rate and intelligibility measures by regressing intelligibility and speaking rate onto the 3-*df* natural cubic splines of age using regular linear models. The residuals of these models provide age-adjusted scores. Following this procedure, speaking rate and intelligibility had correlations that ranged from −.14, *p* = .004, for five-word utterances, to −.03, *p* = .57, for six-word utterances. The positive correlations between speaking rate and intelligibility became negative and smaller in magnitude after adjusting for age in both measures. This result motivates the need for a more thorough analysis.

### Effect of Speaking Rate on Intelligibility

**Figure 2.**
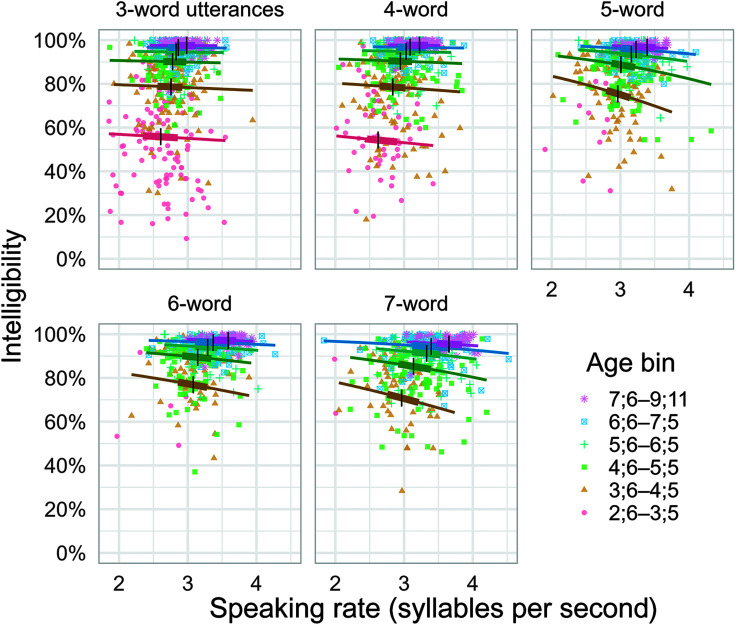
Marginal means of intelligibility by speaking rate, age, and utterance length. Lines represent marginal means (population averages) at focal ages (3;0, 4;0, 5;0, 6;0, 7;0, and 8;0 [years;months]); these are expected intelligibility scores after averaging over between-child variability. Lines contain boxplot landmarks: Vertical marks represent the medians, and thicker intervals contain the middle 50% of the observed data. Points represent observed intelligibility scores. For ease of visualization, children are grouped into 12-month age bins centered around the focal ages (except for the oldest age bin with a focal age of 8;0 and a larger age range due to the sparser sample size after age 7;11). For our analyses, no such age binning was employed.

Figure 2 visualizes the observed intelligibility scores (points) and the model-estimated population-average intelligibility means (lines), and all the key modeling results are represented in the figure.

There was a clear effect of age such that intelligibility increased with age, as documented in Hustad et al.'s (2021) study. This effect is apparent in Figure 2 through the vertical ordering of the regression lines in each panel. For example, in the four-word panel, at 3 syllables per second, the expected intelligibility at age 3;0 was 55% (95% posterior interval [51%, 59%]). The expected intelligibility then increases with age from this baseline: 78% [76%, 80%] at 4;0, 90% [89%, 91%] at 5;0, 95% [94%, 95%] at 6;0, and 97% [96%, 97%] at 7;0.

There was a general trend for the expected intelligibility to decrease with utterance length. For a 4-year-old with a speaking rate of 3 syllables per second, for instance, the expected intelligibility was 78% (95% posterior interval [76%, 80%]) for three-word utterances, 78% [76%, 80%] for four-word utterances, 75% [73%, 77%] for five-word utterances, 77% [75%, 79%] for six-word utterances, and 71% [68%, 73%] for seven-word utterances. For all consecutive changes in utterance length, except for the change from five-word to six-word utterances, there was a decrease or no change in expected intelligibility: four versus three words, 0 [−2, 2]; five versus four words, −3 [−5, −2]; six versus five words, 2 [1, 4]; and seven versus six words, −7 [−8, −5]. These differences, however, marked small changes in intelligibility.

There was also a negative effect of speaking rate such that the expected intelligibility decreased with speaking rate. We again consider the example of a 4-year-old with a speaking rate of 3 syllables per second. An increase in speaking rate of 0.5 syllables per second predicts a corresponding change in expected intelligibility of −1 percentage points (95% posterior interval [−3, 1]) for three-word utterances, −1 [−3, 1] for four-word utterances, −5 [−7, −3] for five-word utterances, −3 [−5, −1] for six-word utterances, and −4 [−6, −2] for seven-word utterances. An increase from 3.0 to 3.5 syllables per second at 6 years old predicts a corresponding change of 0 percentage points [−1, 0] for three-word utterances, 0 [−1, 0] for four-word utterances, −2 [−2, −1] for five-word utterances, −1 [−1, 0] for six-word utterances, and −2 [−2, −1] for seven-word utterances.

#### Within-Child Speaking Rate Effects

To assess whether speaking rate affected intelligibility at the utterance level within children, we fit an additional regression model on a disaggregated subset of the data. We only included data from five- to seven-word utterances (11,282 utterances from 422 children) and ignored any fixed effects of utterance length. We regressed the intelligibility of a single utterance using mixed-effects logistic regression onto the speaking rate of the utterance, the child's average speaking rate on the included utterances, the child's age using splines as in other models, by-child random intercepts (child ability), by-child random slopes of rate (each child has their own rate effect), and by-item random intercept (item easiness).

**Figure 3.**
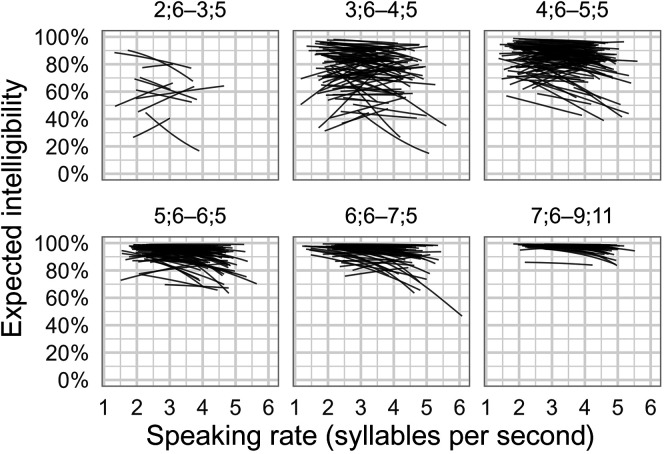
Individual children's estimated speaking rate effects. Each line shows a child's expected intelligibility (posterior mean) along the range of their observed speaking rate. Children are grouped into age bins. Most children follow a negative slope where faster utterances are expected to have reduced intelligibility.

On the model scale (logits, or log-odds of intelligibility), there was a negative effect of speaking rate for a typical child on a typical item such that an increase of speaking rate of 1 syllable per second predicted a corresponding decrease in intelligibility of −0.23 logits (95% posterior interval [−0.30, −0.16]). For 74.6% of participants (315), the child-specific estimated (posterior mean) effect of speaking rate was negative. Figure 3 depicts individual children's model-estimated intelligibility scores (posterior means) along with each child's observed speaking rates. As stated earlier, most children showed a negative effect of speaking rate on intelligibility, but on the outcome scale, the magnitude of this effect was compressed by ceiling effects in older ages. Some children showed a positive relationship between speaking rate and intelligibility; five such children are visible in the first panel.

## Discussion

In this research article, we used data from two previous articles (Hustad et al., 2021; Mahr et al., 2021) that examined speech intelligibility development and articulation rate development, respectively, in typically developing children who ranged in age from 2;9 to 9;11. Here, we combined these data sets on the same children to examine the intersection of speech intelligibility and speech rate with the specific goal of quantifying the relationship between speech intelligibility and speech rate by age in children and to examine the extent to which speech rate affected speech intelligibility. Of interest was whether typically developing children who spoke relatively quickly for their age were less intelligible than their same-age peers and whether typically developing children demonstrated a speed–accuracy trade-off in their habitual speech.

Our analysis strategy followed a path of increasing specificity. First, we looked at a simple relationship between speaking rate and intelligibility and whether that relationship held when we controlled for age. Then, we examined a more rigorous model that incorporated the effects of age, utterance length, and Speaking Rate × Utterance Length, plus child-specific adjustments (i.e., random intercepts). This analysis examined whether utterance length affected intelligibility and whether habitual speaking rate affected intelligibility at the between-child level. That is, are faster children less intelligible than slower children at a given age? Finally, we estimated the effect of speaking rate at the child level (i.e., random slopes) to examine whether faster utterances were less intelligible within an individual child.

We first considered two sets of correlations between intelligibility and speaking rate. We examined the simple Pearson correlation, finding small–moderate positive associations between the two variables where faster speaking rates were associated with higher intelligibility scores. This positive correlation reflected an age confounding, and after adjusting for age, we found null–small negative associations between speech rate and intelligibility. It is possible to reconcile these two correlations from a speech-motor perspective where speech-motor control is a latent skill or ability that develops with age (positive association) and articulation follows speed–accuracy constraints (negative association once age is factored out).

Next, a second set of analyses applied a more rigorous modeling approach that also accounted for utterance length effects. First, we examined the effect of utterance length on intelligibility when controlling for age. Our earlier work suggested a possible length effect as we found that multiword utterances were more intelligible than single-word utterances after 40 months of age (Hustad et al., 2021) but did not include utterance length as its own predictor of intelligibility. The present analysis found a general negative effect of utterance length such that longer utterances tended to be less intelligible than shorter ones. For a typical 4-year-old, the estimated effect of utterance length was a change from 78% intelligibility at three words to 71% at seven words. The effect from length to length was not monotonic; an increase in intelligibility from five- to six-word utterances broke the apparent trend. The estimated utterance length “penalties” were stronger for younger age groups, and this finding can be interpreted as longer utterances placing greater demands on children in this task. Longer utterances require more memory to recall and have greater motor planning and motor execution demands. Maner et al. (2000), for instance, found increased kinematic variability in six-syllable versus 12-syllable utterances in 5-year-olds. Due to the unevenness of the length effect in our sample, however, we regard these utterance length effects foremost as methodological factors that are likely specific to this task and these sentences. Indeed, others applying the TOCS+ have found an uneven effect of length on intelligibility in clinical populations (Darling-White & Polkowitz, 2023).

After examining the effect of utterance length, we next considered the effect of speaking rate within each utterance length while still adjusting for age. The key finding was that intelligibility decreased with increased habitual speaking rate, and this effect was most apparent in longer utterances. Within an utterance length, we found that children who had relatively faster speaking rates had a lower expected intelligibility compared to other children their age. The size of this effect was on the order of 3–5 percentage points (reduced intelligibility) for an increase in speaking rate of 0.5 syllables per second for 4-year-olds in five- to seven-word utterances; for 6-year-olds, the effect diminished to 1–2 percentage points. As a point of reference, these changes in intelligibility (5 percentage points at age 4;0 and 2 percentage points at age 6;0) were approximately the difference between the 50th and 40th intelligibility percentiles at their respective ages based on the model used in our analysis. This habitual rate effect therefore was small at young ages and negligible at older ages.

Because a speed–accuracy trade-off describes a principle of motor control within an individual, we performed an additional analysis that pooled together five- to seven-word utterances and estimated whether individual children showed a negative effect of speaking rate on intelligibility for individual utterances. The model found a negative effect for a statistically typical participant—that is, an average individual on the model's log-odds scale—and indeed, the estimated rate effect was negative for 75% of the observed participants. For the remaining 25% of participants who showed a positive relationship between speaking rate and intelligibility, speaking rate could be indicative of the difficulty of utterance. That is, these children might have made compensatory rate or prosody adjustments (i.e., slowing down on more difficult utterances) or experienced difficult/dysfluent productions.

### Speed–Accuracy Trade-Off

Our working scientific framework for this study has been that articulation follows a speed–accuracy trade-off. That is, more difficult gestures require more time, a relationship observed for individual speakers in Lammert et al.'s (2018) study. Speaking at faster rates reduces the amount of time for articulation, leading speakers to trade accuracy for speed. This relationship is best understood as a description of speech-motor ability within speakers. Indeed, one's habitual speaking rate may not have a bearing on their intelligibility compared to other speakers. Bradlow et al. (1996) found no correlation between intelligibility and average sentence duration for 20 adult speakers, instead finding vowel space area to be a more important predictor of intelligibility.

At the between-speakers level, it is plausible that children who speak more quickly have better speech-motor control. Redford (2014) found children (*n* = 54, aged 5;2–7;11) with faster habitual articulation rates had higher ratings for clarity compared to children with slower articulation rates. The author interprets this effect in terms of speech-motor ability: The children who opt for a faster habitual rate have “superior, more mature articulatory timing control” and thus are perceived to speak more clearly. The finding of a correlation between habitual speaking rate and maximal speaking rate in adults by Tsao and Weismer (1997) is consistent with the idea that speakers use a habitual rate commensurate with their overall speech-motor abilities. (The authors also acknowledge socioindexical factors may affect one's speaking rate.)

Redford's (2014) discussion differentiates between internal versus external factors for rate variability. A speaker's default habitual rate is governed by internal factors (speech-motor ability), but a task's requirement to speak more quickly would be an external factor, and this break from the habitual rate would lead to a speaker trading speed for accuracy. Such external factors are myriad: Kleinow and Smith (2006), examining ten 9- to 10-year-olds, found speaking rate to increase in longer versus shorter utterances, decrease for more complex versus less complex sentences, and increase following a cognitively demanding (Stroop) task versus baseline speaking. Our overall correlation of speaking rate and intelligibility (where older children speak more quickly and have higher intelligibility) supports a general speech-motor ability that improves with age (an internal factor). However, at the same time, longer utterances (external factors) promoted a faster speaking rate and imposed additional demands on the speaker. Thus, we found a negative effect of speaking rate on expected intelligibility in general (population means) and for most individual participants in our sample.

Another set of speaker internal factors is a child's own speech-motor strategy. Vick et al. (2012) applied a cluster analysis on kinematic, perceptual, and acoustic speech measures for 3- to 4-year-olds and identified three clusters along two axes: high phoneme accuracy with low measurement variability, high variability, and low accuracy. These clusters may represent different motor profiles or strategies among speakers or different developmental moments within speakers. In a similar manner, the fact that the speed–accuracy effects held for most, but not all, of the speakers in our study might also reflect different profiles of response to the task: Most speakers will follow a default habitual rate that varies with utterance length, and others may manipulate rate to compensate for more difficult utterances or have to pause in longer utterances.

The question of how a speaker moderates their speaking rate is also another matter of individual variability. The DIVA (Directions Into Velocities of Articulators) model of speech production (Guenther, 1995) provides two strategies for adjusting speaking rate, and speakers vary in how much they rely on each strategy. One strategy is manipulating a control signal that in turn increases the velocity of articulatory movements. The other leverages the speaker-inherent speed–accuracy trade-off by manipulating the system's target space of phonemes. By shrinking a phoneme's target, more precise and hence slower movement is required to attain the motor target. Under this strategy, speakers “hyperarticulate, or use a more ‘canonical’ configuration of the vocal tract, when producing a phoneme at a slower rate” (Guenther, 1995). In other words, for DIVA, speakers articulate more slowly by a combination of moving more slowly and speaking more clearly, and these two options (plus a third of option of pausing) provide a framework for understanding the clinical implications of our work.

### Clinical Implications

The important question for clinical practice is whether children can apply the speed–accuracy trade-off to improve intelligibility. Under ideal circumstances, we might instruct children to speak more slowly, and they would slow down—not by merely moving their articulators more slowly but by aiming for more precise articulatory targets, inherently trading speed for accuracy. However, this ideal circumstance requires children (a) to have those more precise motor/sensory targets and (b) to be able to make the movements for more precise articulation. In other words, a child whose reduced intelligibility is the result of limitations in speech perception may not benefit from the instruction to slow down because their perceptual targets are incorrect.

Another potential issue with rate manipulation is that it requires speech-motor control to slow down in a way that promotes intelligibility. A child may opt to slow down, not by aiming for a more precise target but by moving more slowly or elongating movements, and those movements could lead to atypical or uncoordinated productions. Adams et al. (1993) found that adult speakers showed different patterns of kinematic velocity peaks for reduced speaking rates. At a half- or quarter-speed speaking tempo, speakers would break a single smooth movement into a series of smaller movements, perhaps because at this time scale, sensory feedback could influence speech production in the middle of articulation. Thus, a cue to speak slowly that does not also preserve smoothness or naturalness might not improve intelligibility. On the other hand, it is possible that rate manipulation can help intelligibility without directly changing a child's speech-motor targets. Pilot work by our group tested a rate manipulation strategy based on inserting pauses between words and found an increase in intelligibility in slowed speech compared to habitual speech for children with cerebral palsy and reduced intelligibility (Sakash et al., 2020).

The present study also suggests that manipulating utterance length might have knock-on effects for speech intelligibility. Intuitively, we would expect longer utterances to provide extra context for listeners, but this increased length also taxes the child speaker. Therefore, it is possible that shorter utterances might improve intelligibility for some children.

### Limitations and Future Directions

This study did not directly manipulate the speaking rate of children by instructing them to speak more quickly or more slowly. Furthermore, all sentences were elicited in a structured sentence repetition task instead of natural, spontaneous speaking. Thus, our study can only identify a weak form of a speed–accuracy trade-off from the habitual rates of children. We also only considered two dimensions of speech-motor control (intelligibility and rate), but a richer theoretical model about speech-motor development would incorporate other speech-motor measures such as maximal rate. Finally, we set aside pausing and pause behaviors from consideration in this study. We decided to treat speaking rate as an overall measure of speech-motor movement because of its high correlation with articulation rate and because the task here was a sentence repetition task. That said, a more elaborate model of speaking rate would simultaneously incorporate articulation rate and pausing probability to estimate speaking rate and enrich our understanding of young children's speech production.

The effects of rate on speaking performance have been explored in different clinical populations such as those with dysarthria (Dagenais et al., 2006; Palmer & Enderby, 2007; Van Nuffelen et al., 2010) and those who stutter (Blomgren, 2013; Johnson et al., 2023), but work on typically developing children will clarify the mechanisms that give rise to how clinical rate manipulations affect intelligibility. Thus, further work is needed to examine a speed–accuracy effect on intelligibility within typically developing children. Of interest is whether an increased rate leads to reduced intelligibility but, more importantly, whether a reduced speaking rate can improve intelligibility. Given that some speakers may use different strategies for moderating speech and that an extremely reduced rate may lead to instability or variability, it is important to compare different strategies and cues for reducing rate.

## Data Availability Statement

The data sets analyzed during the current study are not publicly available due to human subjects privacy restrictions. Portions of the data used for the analyses presented in this study may be made available upon reasonable request.

## Supplementary Material

10.1044/2024_JSLHR-24-00356SMS1 Supplemental Material S1 Analysis code.
