# Supplementary material for: Speech Development Between 30 and 119 Months in Typical Children III: Interaction Between Speaking Rate and Intelligibility
Source: J Speech Lang Hear Res. 2024 Dec 16;68(1):79–90. doi: 10.1044/2024_JSLHR-24-00356 (PMC11842040; doi:10.1044/2024_JSLHR-24-00356)
Supplement: Supplemental Material S1 [file JSLHR-68-79-s001.pdf]

Supplemental Material S1. Analysis code.

Tristan Mahr

2024-05-20

Data

The first set of analyses considered each child's mean speaking rate and intelligibility for each utterance length.

```
library(tidyverse)
data_model <- targets::tar_read("data_model_anon")
data_model
#> # A tibble: 2,196 × 14
#>    child speaking_sps artic_sps age_months tocs_level length_longest age_bin
#>    <chr>        <dbl>     <dbl>      <dbl>      <dbl>          <dbl>   <dbl>
#>  1 c464          2.71      2.71         30          3              4      36
#>  2 c464          2.55      2.62         30          4              4      36
#>  3 c017          2.04      2.08         30          3              3      36
#>  4 c315          2.03      2.20         30          3              3      36
#>  5 c468          1.87      1.90         30          3              3      36
#>  6 c154          2.25      2.41         30          3              3      36
#>  7 c338          2.59      2.69         31          3              3      36
#>  8 c110          2.69      2.79         31          3              4      36
#>  9 c110          2.66      2.74         31          4              4      36
#> 10 c431          3.30      3.30         31          3              3      36
#> # ℹ 2,186 more rows
#> # ℹ 7 more variables: intelligibility <dbl>, mwi <dbl>, speaking_sps_3 <dbl>,
#> #   artic_sps_3 <dbl>, age_48 <dbl>, tocs_length <fct>, tocs_level_3 <dbl>
```

where

- `child`: child identifier
- `speaking_sps`, `artic_sps`, `speaking_sps_3`, `artic_sps_3`: speaking rate and articulation rate in syllables per second. The `_3` versions have 3 subtracted so that the values are centered at 3 sps.
- `age_months`, `age_bin`, `age_48`: child age in months, rough age group of child (for plotting), age in months minus 48 (so that the values are centered at 48 months)
- `tocs_level`, `tocs_length`, `tocs_level_3`: utterance length as a number, factor, number centered at 3 word.
- `length_longest`: the longest utterance reached by the child.
- `intelligibility`, `mwi`: observed intelligibility and intelligibility compressed into the range .001–.999 for beta regression.

Correlations

```
data_for_cors <- data_model |>
  group_by(tocs_length) |>
  mutate(
```

```
    # age-residualized
    r_intel = residuals(lm(intelligibility ~ splines::ns(age_months, 3))),
    r_rate = residuals(lm(speaking_sps_3 ~ splines::ns(age_months, 3))),
  ) |>
  ungroup()

data_for_cors |>
  select(tocs_length, age_months, intelligibility, speaking_sps, r_intel, r_rate) |>
  group_by(tocs_length) |>
  reframe(
    pick(everything()) |> as.matrix() |> Hmisc::rcorr() |> broom::tidy()
  ) |>
  mutate(
    estimate = round(estimate, 3),
    p.value = scales::label_pvalue()(p.value)
  ) |>
  knitr::kable(align = "rllrrr")
```

tocs_length	column1	column2	estimate	n	p.value
3	intelligibility	age_months	0.688	538	<0.001
3	speaking_sps	age_months	0.330	538	<0.001
3	speaking_sps	intelligibility	0.216	538	<0.001
3	r_intel	age_months	0.000	538	>0.999
3	r_intel	intelligibility	0.571	538	<0.001
3	r_intel	speaking_sps	-0.069	538	0.107
3	r_rate	age_months	0.000	538	>0.999
3	r_rate	intelligibility	-0.042	538	0.329
3	r_rate	speaking_sps	0.942	538	<0.001
3	r_rate	r_intel	-0.074	538	0.087
4	intelligibility	age_months	0.655	478	<0.001
4	speaking_sps	age_months	0.440	478	<0.001
4	speaking_sps	intelligibility	0.281	478	<0.001
4	r_intel	age_months	0.000	478	>0.999
4	r_intel	intelligibility	0.653	478	<0.001
4	r_intel	speaking_sps	-0.071	478	0.120
4	r_rate	age_months	0.000	478	>0.999
4	r_rate	intelligibility	-0.052	478	0.255
4	r_rate	speaking_sps	0.891	478	<0.001
4	r_rate	r_intel	-0.080	478	0.081
5	intelligibility	age_months	0.566	422	<0.001
5	speaking_sps	age_months	0.438	422	<0.001
5	speaking_sps	intelligibility	0.176	422	<0.001
5	r_intel	age_months	0.000	422	>0.999
5	r_intel	intelligibility	0.768	422	<0.001
5	r_intel	speaking_sps	-0.126	422	0.010
5	r_rate	age_months	0.000	422	>0.999
5	r_rate	intelligibility	-0.108	422	0.027

tocs_length	column1	column2	estimate	n	p.value
5	r_rate	speaking_sps	0.894	422	<0.001
5	r_rate	r_intel	-0.141	422	0.004
6	intelligibility	age_months	0.514	386	<0.001
6	speaking_sps	age_months	0.466	386	<0.001
6	speaking_sps	intelligibility	0.230	386	<0.001
6	r_intel	age_months	0.000	386	>0.999
6	r_intel	intelligibility	0.826	386	<0.001
6	r_intel	speaking_sps	-0.026	386	0.616
6	r_rate	age_months	0.000	386	>0.999
6	r_rate	intelligibility	-0.024	386	0.639
6	r_rate	speaking_sps	0.883	386	<0.001
6	r_rate	r_intel	-0.029	386	0.570
7	intelligibility	age_months	0.525	372	<0.001
7	speaking_sps	age_months	0.508	372	<0.001
7	speaking_sps	intelligibility	0.214	372	<0.001
7	r_intel	age_months	0.000	372	>0.999
7	r_intel	intelligibility	0.829	372	<0.001
7	r_intel	speaking_sps	-0.082	372	0.113
7	r_rate	age_months	0.000	372	>0.999
7	r_rate	intelligibility	-0.080	372	0.125
7	r_rate	speaking_sps	0.857	372	<0.001
7	r_rate	r_intel	-0.096	372	0.064

Beta regression model

> Analyses were carried in the R programming language (vers. 4.4.0, R Core Team, 2024). HMC sampling was performed using Stan (vers. 2.34.1, Stan Development Team, 2024) through the brms R package (vers. 2.21.0, Bürkner, 2017) and the cmdstanr backend (vers. 0.7.1, Gabry, Češnovar, & Johnson, 2024). We compared models with approximate leave-one-out cross validation with the loo R package (vers. 2.7.0, Vehtari, Gelman, & Gabry, 2017).

I wrote a custom function for fitting beta regression models. A user passes in the data and a `flavor` which flavor determines which model formula to use. Models were seeded using dates (`seed`) for reproducibility.

```
library(splines)
library(brms)
fit_beta_model <- function(
  data,
  flavor = NULL,
  priors = NULL,
  formula = NULL,
  file = NULL,
  seed = 20221013,
  adapt_delta = .8,
  iter = 2000
```

```
) {
  formulas <- list(
    ri_main_interaction = bf(
      mwi ~ ns(age_48, knots = c(9, 25), Boundary.knots = c(-18, 71)) +
        tocs_length +
        speaking_sps_3 +
        speaking_sps_3:tocs_length +
        (1 | child),
      phi ~ ns(age_48, knots = 17, Boundary.knots = c(-18, 71)),
      family = Beta()
    ),
    ri_no_main_length = bf(
      mwi ~ ns(age_48, knots = c(9, 25), Boundary.knots = c(-18, 71)) +
        speaking_sps +
        (1 | child),
      phi ~ ns(age_48, knots = 17, Boundary.knots = c(-18, 71)),
      family = Beta()
    ),
    ri_no_main_interaction = bf(
      mwi ~ ns(age_48, knots = c(9, 25), Boundary.knots = c(-18, 71)) +
        tocs_length +
        speaking_sps_3 +
        (1 | child),
      phi ~ ns(age_48, knots = 17, Boundary.knots = c(-18, 71)),
      family = Beta()
    ),
    rs_for_rate = bf(
      mwi ~ ns(age_48, knots = c(9, 25), Boundary.knots = c(-18, 71)) +
        tocs_length +
        speaking_sps_3 +
        speaking_sps_3:tocs_length +
        (speaking_sps_3 | child),
      phi ~ ns(age_48, knots = 17, Boundary.knots = c(-18, 71)),
      family = Beta()
    ),
    rs_monotonic_length = bf(
      mwi ~ ns(age_48, knots = c(9, 25), Boundary.knots = c(-18, 71)) +
        mo(tocs_level) * speaking_sps_3 +
        (mo(tocs_level) | child),
      phi ~ ns(age_48, knots = 17, Boundary.knots = c(-18, 71)),
      family = Beta()
    ),
    rs_for_length = bf(
      mwi ~ ns(age_48, knots = c(9, 25), Boundary.knots = c(-18, 71)) +
        tocs_length +
        speaking_sps_3 +
        speaking_sps_3:tocs_length +
        (tocs_length | child),
      phi ~ ns(age_48, knots = 17, Boundary.knots = c(-18, 71)),
      family = Beta()
    ),
    rs_for_length_plus_rate = bf(
      mwi ~ ns(age_48, knots = c(9, 25), Boundary.knots = c(-18, 71)) +
        tocs_length +
        speaking_sps_3 +
        speaking_sps_3:tocs_length +
        (speaking_sps_3 + tocs_length | child),
      phi ~ ns(age_48, knots = 17, Boundary.knots = c(-18, 71)),
      family = Beta()
    ),
    rs_for_length_colon_rate = bf(
```

```
    mwi ~ ns(age_48, knots = c(9, 25), Boundary.knots = c(-18, 71)) +
      tocs_length +
      speaking_sps_3 +
      speaking_sps_3:tocs_length +
      (speaking_sps_3:tocs_length | child),
    phi ~ ns(age_48, knots = 17, Boundary.knots = c(-18, 71)),
    family = Beta()
  ),
  rs_for_length_colon_rate_no_intercept = bf(
    mwi ~ ns(age_48, knots = c(9, 25), Boundary.knots = c(-18, 71)) +
      tocs_length +
      speaking_sps_3 +
      speaking_sps_3:tocs_length +
      (0 + speaking_sps_3:tocs_length | child),
    phi ~ ns(age_48, knots = 17, Boundary.knots = c(-18, 71)),
    family = Beta()
  ),
  rs_for_length_by_rate = bf(
    mwi ~ ns(age_48, knots = c(9, 25), Boundary.knots = c(-18, 71)) +
      tocs_length +
      speaking_sps_3 +
      speaking_sps_3:tocs_length +
      (speaking_sps_3 * tocs_length | child),
    phi ~ ns(age_48, knots = 17, Boundary.knots = c(-18, 71)),
    family = Beta()
  )
)

files <- c(
  ri_main_interaction = "models/ri_main_interaction",
  ri_no_main_length = "models/ri_no_main_length",
  ri_no_main_interaction = "models/ri_no_main_interaction",
  rs_for_rate = "models/rs_for_rate",
  rs_for_length = "models/rs_for_length",
  rs_for_length_plus_rate = "models/rs_for_length_plus_rate",
  rs_for_length_colon_rate = "models/rs_for_length_colon_rate",
  rs_for_length_colon_rate_no_intercept = "models/rs_for_length_colon_rate_no_intercept",
  rs_for_length_by_rate = "models/rs_for_length_by_rate",
  rs_monotonic_length = "models/rs_monotonic_length"
)

formula <- formula %||% formulas[[flavor]]
file <- file %||% files[[flavor]]


if (is.null(priors)) {
  priors <- c(
    set_prior("normal(0, 2.5)", class = "b"),
    set_prior("normal(0, 1)", class = "sd")
  )
  has_element <- function(x, y) any(x %in% y)
  has_correlation <- get_prior(formula, data = data) |>
    getElement("class") |>
    has_element("cor")
  if (has_correlation) {
    priors <- c(
      priors,
      set_prior("lkj(2)", class = "cor")
    )
  }
```

```
  }


  t <- brm(
    formula = formula,
    data = data,
    prior = priors,
    backend = "cmdstanr",
    seed = seed,
    file = file,
    file_refit = "on_change",
    chains = 4,
    cores = 4,
    iter = iter,
    save_pars = save_pars(all = TRUE),
    control = list(adapt_delta = adapt_delta),
    refresh = 50
  )
  add_criterion(t, c("loo", "loo_R2"))
}
```

The models compared with in the manuscript were as follows:

```
loo_comparison <- targets::tar_read("loo_comparison")
labels <- tibble::tribble(
  ~model, ~random_effects,
  "model_rs_length", "1 + length | child",
  "model_rs_rate", "1 + rate | child",
  "model_rs_length_plus_rate", "1 + length + rate | child",
  "model_main", "1 | child",
  "model_rs_length_colon_rate", "1 + length:rate | child",
  "model_rs_monotonic_length", "1 + monotonic-length | child",
)

loo_table_data <- loo_comparison |>
  inner_join(labels, by = "model") |>
  mutate(
    across(c(2:3), \(x) round(x, 1)),
    across(c(4:9), \(x) round(x, 0)),
    across(c(loo_r2_mean), \(x) round(x, 2)),
    across(c(loo_r2_sd), \(x) round(x, 3)),
  ) |>
  relocate(model, random_effects)

loo_table_data |>
  knitr::kable()
```

Supplemental material, Mahr et al., “Speech Development Between 30 and 119 Months in Typical Children III: Interaction Between Speaking Rate and Intelligibility,” *JSLHR*, https://doi.org/10.1044/2024_JSLHR-24-00356

model	random_effects	elpd_diff	se_diff	elpd_loo	se_elpd_loo	p_loo	se_p_loo	looic	se_looic	loo_r2_mean	loo_r2_sd
model_rs_length	1 + length | child	0.0	0.0	3814	49	852	20	-7628	98	0.84	0.009
model_rs_length_plus_rate	1 + length + rate | child	-28.8	5.3	3785	50	784	20	-7571	100	0.82	0.010
model_rs_monotonic_length	1 + monotonic-length | child	-98.9	9.1	3715	51	523	16	-7431	102	0.79	0.011
model_rs_rate	1 + rate | child	-101.4	9.8	3713	52	490	15	-7426	104	0.78	0.011
model_main	1 | child	-107.2	9.7	3707	52	442	15	-7414	104	0.78	0.011
model_rs_length_colon_rate	1 + length:rate | child	-107.9	9.8	3706	52	479	15	-7413	104	0.78	0.011

All models (except `model_rs_monotonic_length`) formula includes a categorical effect of utterance length `tocs_length`, speaking rate centered at 3 sps `tocs_length`, an adjustment to the rate effect for each length `speaing_sps_3:tocs_length`, and the 3-df spline for age `ns(...)`. The `phi ~` regression lines allows the dispersion of the beta distribution to change with age. What differentiated these models were the variables used in the random (by-child) effects. `model_rs_monotonic_length` `model_rs_monotonic_length` model applies the method in Bürkner & Charpentier (2020), using `mo()` to mark a variable as monotonic.

The winning model on the basis of model comparison was `model_rs_length` (by-child length effects).

```
model_rs_length <- targets::tar_read(model_rs_length)
summary(model_rs_length)
#>  Family: beta
#>   Links: mu = logit; phi = log
#> Formula: mwi ~ ns(age_48, knots = c(9, 25), Boundary.knots = c(-18, 71)) + tocs_length + speaking_sps_3 + speaking_sps_3:tocs_length + (tocs_length | child)
#>          phi ~ ns(age_48, knots = 17, Boundary.knots = c(-18, 71))
#>    Data: data (Number of observations: 2196)
#>   Draws: 4 chains, each with iter = 3000; warmup = 1500; thin = 1;
#>          total post-warmup draws = 6000
#>
#> Multilevel Hyperparameters:
#> ~child (Number of levels: 538)
#>                                  Estimate Est.Error l-95% CI u-95% CI Rhat
#> sd(Intercept)                        0.80      0.04     0.71     0.88 1.01
#> sd(tocs_length4)                     0.50      0.09     0.28     0.65 1.02
#> sd(tocs_length5)                     0.38      0.11     0.11     0.55 1.02
#> sd(tocs_length6)                     0.44      0.09     0.22     0.59 1.02
#> sd(tocs_length7)                     0.54      0.09     0.34     0.68 1.02
#> cor(Intercept,tocs_length4)         -0.27      0.10    -0.43    -0.02 1.01
#> cor(Intercept,tocs_length5)         -0.45      0.10    -0.62    -0.23 1.00
#> cor(tocs_length4,tocs_length5)       0.41      0.18    -0.08     0.66 1.01
#> cor(Intercept,tocs_length6)         -0.31      0.13    -0.49    -0.01 1.00
#> cor(tocs_length4,tocs_length6)       0.57      0.13     0.26     0.77 1.00
#> cor(tocs_length5,tocs_length6)       0.53      0.19     0.01     0.77 1.01
#> cor(Intercept,tocs_length7)         -0.26      0.12    -0.44     0.03 1.01
#> cor(tocs_length4,tocs_length7)       0.54      0.12     0.25     0.73 1.00
#> cor(tocs_length5,tocs_length7)       0.45      0.18    -0.03     0.70 1.01
#> cor(tocs_length6,tocs_length7)       0.86      0.10     0.67     0.96 1.01
#>                                  Bulk_ESS Tail_ESS
#> sd(Intercept)                         191      280
#> sd(tocs_length4)                      120      163
#> sd(tocs_length5)                      125      181
#> sd(tocs_length6)                      136      160
#> sd(tocs_length7)                      144      163
#> cor(Intercept,tocs_length4)           290      306
#> cor(Intercept,tocs_length5)          2818      915
#> cor(tocs_length4,tocs_length5)        369      270
#> cor(Intercept,tocs_length6)           380      273
#> cor(tocs_length4,tocs_length6)        662      480
#> cor(tocs_length5,tocs_length6)        423      248
#> cor(Intercept,tocs_length7)           232      239
#> cor(tocs_length4,tocs_length7)        718      463
#> cor(tocs_length5,tocs_length7)        384      241
#> cor(tocs_length6,tocs_length7)        567      280
```

```
#>
#> Regression Coefficients:
#>                                              Estimate Est.Error l-95% CI
#> Intercept                                       -0.42      0.11    -0.64
#> phi_Intercept                                    3.82      0.39     3.08
#> nsage_48knotsEQc925Boundary.knotsEQcM18711       3.25      0.15     2.95
#> nsage_48knotsEQc925Boundary.knotsEQcM18712       6.80      0.28     6.26
#> nsage_48knotsEQc925Boundary.knotsEQcM18713       3.63      0.21     3.22
#> tocs_length4                                    -0.10      0.05    -0.20
#> tocs_length5                                    -0.31      0.05    -0.41
#> tocs_length6                                    -0.17      0.06    -0.28
#> tocs_length7                                    -0.51      0.06    -0.63
#> speaking_sps_3                                  -0.08      0.11    -0.31
#> tocs_length4:speaking_sps_3                     -0.05      0.13    -0.30
#> tocs_length5:speaking_sps_3                     -0.45      0.12    -0.69
#> tocs_length6:speaking_sps_3                     -0.25      0.13    -0.51
#> tocs_length7:speaking_sps_3                     -0.34      0.13    -0.59
#> phi_nsage_48knotsEQ17Boundary.knotsEQcM18711     0.13      0.67    -1.22
#> phi_nsage_48knotsEQ17Boundary.knotsEQcM18712     0.53      0.23     0.07
#>                                              u-95% CI Rhat Bulk_ESS Tail_ESS
#> Intercept                                       -0.19 1.00     2038     3248
#> phi_Intercept                                    4.59 1.02      118      212
#> nsage_48knotsEQc925Boundary.knotsEQcM18711       3.55 1.00     2596     3175
#> nsage_48knotsEQc925Boundary.knotsEQcM18712       7.35 1.00     1873     3560
#> nsage_48knotsEQc925Boundary.knotsEQcM18713       4.03 1.00     3657     3703
#> tocs_length4                                    -0.01 1.00     3297     3803
#> tocs_length5                                    -0.22 1.00     3023     4015
#> tocs_length6                                    -0.06 1.00     3206     4399
#> tocs_length7                                    -0.41 1.00     2993     3682
#> speaking_sps_3                                   0.14 1.00     1971     2564
#> tocs_length4:speaking_sps_3                      0.21 1.00     3431     4386
#> tocs_length5:speaking_sps_3                     -0.21 1.00     3030     4074
#> tocs_length6:speaking_sps_3                      0.01 1.00     3197     3824
#> tocs_length7:speaking_sps_3                     -0.08 1.00     2527     3713
#> phi_nsage_48knotsEQ17Boundary.knotsEQcM18711     1.33 1.02      140      352
#> phi_nsage_48knotsEQ17Boundary.knotsEQcM18712     0.97 1.01      290      843
#>
#> Draws were sampled using sample(hmc). For each parameter, Bulk_ESS
#> and Tail_ESS are effective sample size measures, and Rhat is the potential
#> scale reduction factor on split chains (at convergence, Rhat = 1).
```

However, it bears noting that a more appropriate model comparison would compare them using leave-one-child-out cross-validation. This approach was computational prohibitive because it would require fitting 538 versions of each model.

Marginal means

With a model in hand, we compute marginal predictions by simulating new children from each posterior sample. The basic recipe is as follows.

Create a grid of data to make predictions for, giving `child` an out-of-sample value. To make things easier, I break the data into batches in a list.

```
newdata <- tidyr::crossing(
  age_48 = (3:7) * 12 - 48,
  tocs_length = c("3", "4", "5", "6", "7"),
  speaking_sps_3 = c(0, .5),
  child = "fake"
```

```
)
newdata
#> # A tibble: 50 × 4
#>    age_48 tocs_length speaking_sps_3 child
#>     <dbl> <chr>                <dbl> <chr>
#>  1    -12 3                      0   fake
#>  2    -12 3                      0.5 fake
#>  3    -12 4                      0   fake
#>  4    -12 4                      0.5 fake
#>  5    -12 5                      0   fake
#>  6    -12 5                      0.5 fake
#>  7    -12 6                      0   fake
#>  8    -12 6                      0.5 fake
#>  9    -12 7                      0   fake
#> 10    -12 7                      0.5 fake
#> # ℹ 40 more rows


newdata_batches <- newdata |>
  split(~ age_48:speaking_sps_3)
```

Next I sample 1000 children from each draw. The 1000 childen are 1000 draws from the multivariate normal distribution used in the random effects. First I extract the variance-covariance matrix of the random effects. I use an `rvar()` which allows me to write code for the 6000 x 5 x 5 array of posterior draws as if it were a 5 x 5 matrix. The random effects are centered at 0 so each row of newdata is given a mean of 0.

```
cov <- model_rs_length |>
  VarCorr(summary = FALSE) |>
  _$child$cov |>
  posterior::rvar()
cov
#> rvar<6000>[5,5] mean ± sd:
#>              Intercept      tocs_length4   tocs_length5   tocs_length6  
#> Intercept     0.64 ± 0.071  -0.11 ± 0.054  -0.14 ± 0.055  -0.11 ± 0.056
#> tocs_length4 -0.11 ± 0.054   0.26 ± 0.084   0.09 ± 0.051   0.13 ± 0.056
#> tocs_length5 -0.14 ± 0.055   0.09 ± 0.051   0.16 ± 0.075   0.10 ± 0.052
#> tocs_length6 -0.11 ± 0.056   0.13 ± 0.056   0.10 ± 0.052   0.20 ± 0.075
#> tocs_length7 -0.12 ± 0.062   0.15 ± 0.060   0.10 ± 0.055   0.21 ± 0.070
#>              tocs_length7  
#> Intercept    -0.12 ± 0.062
#> tocs_length4  0.15 ± 0.060
#> tocs_length5  0.10 ± 0.055
#> tocs_length6  0.21 ± 0.070
#> tocs_length7  0.30 ± 0.087


means <- rep(0, ncol(cov))

rows_batches <- vapply(newdata_batches, nrow, integer(1))
stopifnot(all(rows_batches == length(means)))

num_children <- 1000
sim_children <- posterior::rdo(mvtnorm::rmvnorm(num_children, means, cov))
```

Given the simulated children, I walk through each batch of new data, get the linear predictions (predictions in logits using just the fixed effects), add the simulated children to

each linear prediction, convert the logits into proportions and then average over the 1000 children for that draw.

```
l <- newdata_batches |>
  lapply(tidybayes::add_linpred_rvars, model_rs_length, re_formula = NA) |>
  lapply(
    function(x) {
      x$.marginal <- (sim_children + t(x$.linpred)) |>
        brms::inv_logit_scaled() |>
        posterior::rvar_apply(2, posterior::rvar_mean)
      x
    }
  ) |>
  bind_rows()
```

This part is time-consuming so I load in the precomputed value.

```
targets::tar_read(marginal_means_model_rs_length)
#> # A tibble: 50 × 6
#>    age_48 tocs_length speaking_sps_3 child        .linpred     .marginal
#>     <dbl> <chr>                <dbl> <chr>       <rvar[1d]>    <rvar[1d]>
#>  1    -12 3                        0 fake    0.231 ± 0.080  0.55 ± 0.018
#>  2    -12 4                        0 fake    0.130 ± 0.080  0.53 ± 0.019
#>  3    -12 5                        0 fake   -0.078 ± 0.077  0.48 ± 0.019
#>  4    -12 6                        0 fake    0.061 ± 0.082  0.51 ± 0.020
#>  5    -12 7                        0 fake   -0.283 ± 0.084  0.43 ± 0.020
#>  6      0 3                        0 fake    1.448 ± 0.065  0.78 ± 0.011
#>  7      0 4                        0 fake    1.347 ± 0.063  0.78 ± 0.010
#>  8      0 5                        0 fake    1.138 ± 0.058  0.75 ± 0.011
#>  9      0 6                        0 fake    1.278 ± 0.064  0.77 ± 0.011
#> 10      0 7                        0 fake    0.933 ± 0.066  0.71 ± 0.013
#> # ℹ 40 more rows
```

The `.marginal` column has 6000 marginal means for each row of the prediction grid. Subtracting between two rows would give 6000 differences. Estimates and comparisons were computed by taking the median and 95% quantile intervals of the means and differences.

Logistic regression model

For the within-child effects of rate, we use the item-level data for the 5-, 6-, and 7-word utterances.

```
data_model_by_item_anon <- targets::tar_read(data_model_by_item_anon)
data_model_by_item_anon
#> # A tibble: 11,282 × 18
#>    child item  speaking_sps artic_sps age_months tocs_level length_longest
#>    <chr> <chr>        <dbl>     <dbl>      <dbl>      <dbl>          <dbl>
#>  1 c130  S5T01         2.47      2.47         34          5              5
#>  2 c130  S5T02         2.26      2.26         34          5              5
#>  3 c130  S5T03         2.55      2.55         34          5              5
#>  4 c130  S5T04         2.77      2.77         34          5              5
#>  5 c130  S5T05         3.01      3.01         34          5              5
#>  6 c130  S5T06         1.89      2.12         34          5              5
#>  7 c130  S5T07         2.39      2.39         34          5              5
#>  8 c130  S5T08         2.20      2.50         34          5              5
```

```
#>  9 c130  S5T09         2.83      2.83         34          5               5
#> 10 c227  S5T01         3         3            35          5               5
#> # ℹ 11,272 more rows
#> # ℹ 11 more variables: age_bin <dbl>, intelligibility <dbl>,
#> #   n_words_correct <dbl>, n_words_total <dbl>, mwi <dbl>,
#> #   speaking_sps_3 <dbl>, artic_sps_3 <dbl>, age_48 <dbl>, tocs_length <fct>,
#> #   tocs_level_3 <dbl>, child_mean_rate <dbl>
```

New here are

- `item`: item identifier
- `n_words_correct`, `n_words_total`: number of words correctly transcribed by the listeners and number of words total, so the intelligibility is the proportion of words correctly transcribed
- `child_mean_rate`: a child's average speaking rate over all items

The fitted model was the following:

```
model_logistic <- targets::tar_read(
  "model_rs_rate_ri_item_no_length_binom_mundlak"
)
model_logistic
#>  Family: binomial
#>   Links: mu = logit
#> Formula: n_words_correct | trials(n_words_total) ~ ns(age_48, knots = c(9, 25),
Boundary.knots = c(-18, 71)) + speaking_sps_3 + child_mean_rate + (speaking_sps_3 | child) +
(1 | item)
#>    Data: data (Number of observations: 11282)
#>   Draws: 4 chains, each with iter = 3000; warmup = 1500; thin = 1;
#>          total post-warmup draws = 6000
#>
#> Multilevel Hyperparameters:
#> ~child (Number of levels: 422)
#>                               Estimate Est.Error l-95% CI u-95% CI Rhat
#> sd(Intercept)                     0.85      0.03     0.78     0.92 1.00
#> sd(speaking_sps_3)                0.50      0.03     0.44     0.56 1.00
#> cor(Intercept,speaking_sps_3)    -0.15      0.07    -0.28    -0.01 1.00
#>                               Bulk_ESS Tail_ESS
#> sd(Intercept)                     1367     2649
#> sd(speaking_sps_3)                2518     3793
#> cor(Intercept,speaking_sps_3)     1917     2597
#>
#> ~item (Number of levels: 29)
#>               Estimate Est.Error l-95% CI u-95% CI Rhat Bulk_ESS Tail_ESS
#> sd(Intercept)     0.71      0.09     0.55     0.92 1.00     1108     2461
#>
#> Regression Coefficients:
#>                                              Estimate Est.Error l-95% CI u-95% CI
#> Intercept                                        0.49      0.33    -0.16     1.14
#> nsage_48knotsEQc925Boundary.knotsEQcM18711      2.93      0.20     2.52     3.33
#> nsage_48knotsEQc925Boundary.knotsEQcM18712      4.72      0.66     3.42     6.02
#> nsage_48knotsEQc925Boundary.knotsEQcM18713      3.30      0.27     2.74     3.83
#> speaking_sps_3                                  -0.23      0.04    -0.30    -0.16
#> child_mean_rate                                 -0.12      0.15    -0.42     0.17
#>                                              Rhat Bulk_ESS Tail_ESS
#> Intercept                                    1.00      879     1846
#> nsage_48knotsEQc925Boundary.knotsEQcM18711 1.00     1064     1742
#> nsage_48knotsEQc925Boundary.knotsEQcM18712 1.00     1098     1950
```

```
#> nsage_48knotsEQc925Boundary.knotsEQcM18713 1.00     1810     2818
#> speaking_sps_3                             1.00     4260     4387
#> child_mean_rate                            1.00     1031     2004
#>
#> Draws were sampled using sample(hmc). For each parameter, Bulk_ESS
#> and Tail_ESS are effective sample size measures, and Rhat is the potential
#> scale reduction factor on split chains (at convergence, Rhat = 1).
```

The random effects include a by-child random intercept, by-child rate effects, and by-item intercepts. The same age-spline is used as before for the fixed effects. The other two predictors are the speaking rate on the given utterance and the child's average speaking rate. The `child_mean_rate` is meant to absorb child-level reflect a child's habitual rate so that the `speaking_sps_3` can estimate the within child effects of rate.

We can extract the posterior means of each child's rate coefficient like so:

```
# Posterior draws of the slopes
child_slopes <- model_logistic |>
  coef(summary = FALSE) |>
  getElement("child") |>
  _[, , c("Intercept", "speaking_sps_3")] |>
  posterior::rvar()

slope_means <- mean(child_slopes)[, "speaking_sps_3"]

f_percent <- scales::label_percent(.1)
lslopes <- list(
  n_positive = sum(slope_means > 0),
  n_negative = sum(slope_means < 0),
  pct_positive = f_percent(sum(slope_means > 0) / length(slope_means)),
  pct_negative = f_percent(sum(slope_means < 0) / length(slope_means))
)
str(lslopes)
#> List of 4
#>  $ n_positive  : int 107
#>  $ n_negative  : int 315
#>  $ pct_positive: chr "25.4%"
#>  $ pct_negative: chr "74.6%"
```

The plotted lines used `tidybayes::add_epred_rvars()` to get child-level predictions when `trials` is set to 1. We take a grid of 50 points along each child's observed speaking rate, get posterior expectations for each of those point and plot the mean of those expectations for each child.

```
data_binom_rate_grid <- data_model_by_item_anon |>
  group_by(child, age_months, age_bin, age_48) |>
  reframe(
    child_mean_rate = mean(speaking_sps_3),
    speaking_sps_3 = c(
      seq(min(speaking_sps_3), max(speaking_sps_3), length.out = 50),
      child_mean_rate
    ),
    item = "fake",
    n_words_total = 1
  )

data_binom_rate_grid <- tidybayes::add_epred_rvars(
```

```
  data_binom_rate_grid,
  model_logistic,
  allow_new_levels = TRUE
)
```

References

Bürkner, P.-C. (2017). brms: An R package for Bayesian multilevel models using Stan. *Journal of Statistical Software*, *80*(1), 1–28. doi:10.18637/jss.v080.i01

Bürkner, P.-C., & Charpentier, E. (2020). Modelling monotonic effects of ordinal predictors in Bayesian regression models. *British Journal of Mathematical and Statistical Psychology*, *73*(3), 420–451. doi:10.1111/bmsp.12195

Gabry, J., Češnovar, R., & Johnson, A. (2024). *cmdstanr: R interface to CmdStan*. Retrieved from https://mc-stan.org/cmdstanr/

R Core Team. (2024). *R: A language and environment for statistical computing*. Vienna, Austria: R Foundation for Statistical Computing. Retrieved from https://www.R-project.org/

Stan Development Team. (2024). *Stan modeling language users guide and reference manual*. Retrieved from https://mc-stan.org

Vehtari, A., Gelman, A., & Gabry, J. (2017). Practical Bayesian model evaluation using leave-one-out cross-validation and WAIC. *Statistics and Computing*, *27*, 1413–1432. doi:10.1007/s11222-016-9696-4
